# On the restricted three-body model of the dynamical behaviour of masses of *C*_70_ fullerenes at the nanoscale

**DOI:** 10.1016/j.heliyon.2022.e08899

**Published:** 2022-02-03

**Authors:** Jagadish Singh, Richard Kanshio Tyokyaa

**Affiliations:** a Department of Mathematics, Faculty of Physical Sciences, Ahmadu Bello University, Zaria, Nigeria; b Department of Mathematical Sciences, Faculty of Physical Sciences, Federal University Dutsin-Ma, P.M.B 5001 Dutsin-Ma, Katsina State, Nigeria

**Keywords:** Restricted three-body problem, $C_{70}$ fullerenes, Nanoscale, Positions, Stability

## Abstract

The present study investigates the positions and stability of $C_{70}$ fullerenes. Numerically, the positions and stability have been computed $C_{70}$ fullerene at the nanoscale to show the effects of the parameters involved with the help of software MATHEMATICA. The total molecular energy evidenced refers to the total pairwise molecular interactions between two C 70 fullerenes, which is an approximation of a continuous approach. The attractive Vand der Waals forces between only molecules provide the centripetal forces between three fullerenes. The total pairwise molecular interactions between two C 70 fullerenes, which is an approximation of a continuous approach, is termed the total molecular energy evidenced. The attractive Vand der Waals forces between single molecules create the centripetal forces between three fullerenes. We have predicted the collective potential function, mutual force and angular velocity. The stationary points are collinearly lying on the ξ−axis which are symmetric about the η−axis as the molecules of the carbon atoms in the nucleus are evenly distributed. There is at least one complex root with the positive real part for each set of values, it has been discovered. The stationary points are thus unstable in the Lyapunov sense.

## 1 General introduction

The restricted three-body problem (R3BP) is a problem that has continues to be of great theoretical, practical, historical and educational importance. The study of this problem has had important implications in various scientific fields, some of which includes; celestial mechanics chaos theory, galactic dynamics, molecular physics and many others. This problem is still a stimulating and active research field that is receiving considerable attention of scientists and astronomers due to its applications in dynamics of the stellar and solar systems, artificial satellites and lunar theory.

Many studies have been carried out in the field of celestial mechanics. Shalini K., et al. [1] examined the Restricted Three-body Problem in the non-linear sense, the triangular when the smaller primary is a heterogeneous spheroid with different densities. They confirmed that in the non-linear sense, the triangular libration point $L_{4}$ is stable in the range $0<\mu <\mu_{c}$ except $\mu_{1}^{\prime }$, $\mu_{2}^{\prime }$, $\mu_{3}^{\prime }$ at which Moser's theorem is not applicable. In general, the shapes of the celestial bodies in the R3BP are assumed to be spherical. However, some notable researchers [2], [3], have observed the significant effects of oblateness of the bodies. Suraj et al. [4] considered the out-of-plane equilibrium points in the planar Restricted Three-body Problem with heterogeneous oblate primaries and three layers of different densities. They confirmed that the points are unstable for all mass ratios and other parameters.

It is well known that nature possesses certain phenomena that establish similarity and order in their co-existence. These observed phenomena have direct links to models that are found in science and technology. Most inventions of modern days' technologies are designed after nature. For instance, flying machines such as aeroplanes, jet fighters, etc., which function on aerodynamics law, are designed from keen observation of birds. Swarm robotics is designed after the flocking behaviour of birds and insects. More so, petals of flowers appear precisely in the Fibonacci sequence.

In like manner, when we observe on a large scale the configuration of our solar system and other exoplanets, we discover a high correlation in their dynamic behaviour with occurrences at the nanoscale sub-atomic level. In a nutshell, we know Kepler's first law, which stipulates that all planets revolve around the sun in an elliptic orbit. The same phenomenon applies in the atomic realm, where all the electrons revolve around the nucleus. It then implies that the orbital motion is a common phenomenon of dynamics at mega and nanoscale levels.

In addition to the above, motions of elements at both the mega-scale and nano-scale levels are governed by the same principles. For instance, the gravitational attraction between two-point masses undergoing planetary motion is driven by the inverse square law. Likewise, the inverse square law applies to the motion of electrons at the sub-atomic level. Considering these staking similarities, it is of keen scientific interest to investigate the dynamical behaviour of masses at the sub-atomic level. To this effect, we shall employ the restricted three-body model to undergo this study using $C_{70}$ fullerenes as a case study.

Kroto et al. [5] were the first to discover Fullerenes. Buckminsterfullerene was named after Buckminster Fuller, the architect who designed the Geodesic Domes in the 1960s. They have icosahedral symmetry and are made up of 20 hexagonal and 12 pentagonal rings structurally. The behaviour of $C_{70}$ fullerene seems like electron-deficient Alkenes and reacts rich with electron's species. The high symmetry exhibited by $C_{70}$ molecule has made it the unique property of the group. Carbon 70 fullerene's rotation around an axis in a plane has 120 symmetry operations which enable the mapping of its molecule onto itself, which in turn makes $C_{70}$ like $C_{60}$ the molecule with the most significant number of symmetry operations and the most symmetric molecules [6].

Structurally, $C_{70}$ fullerene is made up of 70 carbon atoms. The fullerene appears to be a rugby ball made up of 25 hexagons and 12 pentagons in a cage-like fused-ring structure, with a bond along each polygon edge with a carbon atom at the vertices of each polygon. Its structure is similar to that of C_60_ molecule (20 hexagons and 12 pentagons), but it has a belt of 5 hexagons inserted at the equator.

Fullerenes and carbon nanotubes have attracted attention, specifically in creating high-frequency nanoscale oscillators or gigahertz oscillators for ultrafast optical filters and nano-antennae applications. Fullerenes, being allotropes of carbon, have been considered a new class of molecules. Unlike diamond and graphite, they are made up of a hollow carbon cage structure. Research in fullerenes has resulted in the synthesis of more than a thousand new compounds. Fullerenes' discovery has been the object of interest to scientists all over the world. Fullerenes provide abundant research opportunities in pure chemistry, material science, pharmaceutical chemistry, nanotechnology, etc.

Kroto [7] studied the stability of fullerenes $C_{n}$ with n=24,28,32,36,50,60, *and* 70. He concluded that Huckel's rule gives aromaticity in spherical systems than planar systems. $C_{70}$ and $C_{60}$ were found to be more aromatic and highly stable than that of $C_{24},C_{28},\ldots C_{50}$. The high stability of these fullerenes is due to the reduced strain in the ring as the twelve pentagons are isolated from each other. Taylor [8] further applied the Huckel rule to different fullerene derivatives like $C_{50},C_{60},C_{70},C_{80}$ and $C_{84}$ and observed that only $C_{50}$ and $C_{70}$ were predicted to be more aromatic and $C_{60}$ was less aromatic.

Chan et al. [9] conducted a research of two-body problems at the nanoscale, including fullerene-fullerene and fullerene-carbon nanotube. Chan et al. [10] looked at the placements of three C 60 fullerenes, a carbon atom, and two C 60 fullerenes in the circular planar constrained three-body issue. The maximum angular frequency of two and three fullerene systems reaches the terahertz range, according to their findings. Cami et al. [11] confirmed that the spectral signatures of $C_{60}$ and $C_{70}$ were observed by NASA's Spitzer Infrared Telescope in a cloud of Cosmic dust surrounding a star 6500 light-years away. The recent studies of [12] and [13] show that the ionized $C_{60}$ and $C_{70}$ molecules were detected with the Hubble Space Telescope in the space between those stars.

In our study, we wish to examine the classical restricted three-body problem for three carbon 70 fullerenes ($C_{70}$ fullerenes) at the nanoscale, neglecting any thermal fluctuations arising from the environment.

The paper spans 5 sections: Section 2 outlines the methods used for the problem under review. The study results are presented in section 3, while the discussions and conclusion are presented in sections 4 and 5 respectively.

## 2 Equations of motion

In this section, we considered the Lennard-Jones potential energy for an offset atom and an offset-fullerenes where carbon atoms are assumed to be uniformly distributed over the surface of molecules and have adopted the classical [14] potential as our potential function.

The non-bonded interaction energy can be derived via continuous approximation using the Lennard-Jones potential energy for an offset atom and an offset fullerene, where carbon atoms are assumed to be equally distributed over the surface of molecules. That is:

(1) $$E=n_{g}n_{f}\int_{\Sigma_{g}}\int_{\Sigma_{f}}\Phi (r)d_{\Sigma_{f}}d_{\Sigma_{g}}.$$

Where $n_{g}$ and $n_{f}$ represent the mean surface density of carbon atoms on a carbon nanotube and a fullerene respectively, and *r* denotes the distance between two typical surface elements $d_{\Sigma_{g}}$ and $d_{\Sigma_{f}}$ on the two different molecules. We have adopted the classical [14] potential as our potential function given as;

(2) $$\Phi (r)=4\varepsilon \left[{\left(\frac{\varphi }{\rho }\right)}^{12}-{\left(\frac{\varphi }{\rho }\right)}^{6}\right]=-\frac{A}{\rho^{6}}+\frac{B}{\rho^{12}}.$$

Where ρ,ε,φ,A *and B* is the distance between two arbitrary carbon atoms, the potential well depth of the two carbon atoms, the parameter determined by the equilibrium distance, and the attractive and the attractive repulsive constant.

Considering equations (1), (2) and following the derivation by [15], we obtain

(3) $$\Phi \left(r\right)=-\Psi_{6}\left(r\right)+\Psi_{12}(r).$$

Where $\Psi_{n}$ is defined by

(4) $$\Psi_{n}\left(r\right)=\frac{4\pi^{2}a^{2}C_{n}n_{g}n_{f}}{r\left(n-2\right)\left(n-3\right)}(\frac{1}{{\left(2a+r\right)}^{n-3}}-\frac{1}{{\left(2a-r\right)}^{n-3}}-\frac{2}{r^{n-3}}).$$

Where $a,C_{6}$ and $C_{12}$ are the radius of the $C_{70}$ fullerene, *A* and *B* respectively.

Consider the three-body problem at the nanoscale, where fullerene centres are positioned at the vertices of an equilateral triangle. Then, the mutual force F(r) between any two fullerenes can be given as

(5) $$F\left(r\right)=-\frac{d\Phi \left(r\right)}{dr}.$$

The force exerted on a fullerene by the other two fullerenes is given by Newton's second law of motion:

(6) $$m_{k}\overset{¨}{\overset{\to }{r_{k}}}=\sum_{j\neq k=1}^{3}F(r_{kj})\frac{\overset{\to }{r_{j}}-\overset{\to }{r_{k}}}{r_{kj}}.$$

The total mass of the *k*−fullerene, the position vector of the *k*−fullerene and the relative displacement of any two fullerenes *k* and *j* are respectively represented as $m_{k}$, $r_{k}$, and $r_{kj}$.

We let $\overset{\to }{r}=\overset{\to }{r_{2}}-\overset{\to }{r_{1}}$, $\overset{\to }{R}$ to be the centre of mass of $m_{1}$ and $m_{2}$ allocated at the origin of the coordinate system and $\overset{\to }{\rho }=\overset{\to }{r_{3}}-\overset{\to }{R}=M\mu^{-1}\overset{\to }{r_{3}}$ where $\mu =m_{1}+m_{2}$. With these assumptions, we have;

$$\overset{\to }{r_{2}}-\overset{\to }{r_{1}}=\overset{\to }{r},$$

(7) $$\overset{\to }{r_{3}}-\overset{\to }{r_{1}}=\overset{\to }{\rho }+m_{2}\mu^{-1}\overset{\to }{r},$$

$$\overset{\to }{r_{3}}-\overset{\to }{r_{2}}=\overset{\to }{\rho }-m_{1}\mu^{-1}\overset{\to }{r}.$$

The Jacobian coordinates $\overset{\to }{r}$ and $\overset{\to }{\rho }$ given in terms of equations of motion are then given by

(8) $$\overset{¨}{\overset{\to }{r}}=\frac{-\mu }{m_{1}m_{2}}\frac{F(r_{12})\overset{\to }{r}}{r_{12}}+\frac{1}{m_{2}}\frac{F\left(r_{23}\right)}{r_{23}}\left(\overset{\to }{\rho }-m_{1}\mu^{-1}\overset{\to }{r}\right)-\frac{1}{m_{1}}\frac{F\left(r_{13}\right)}{r_{13}}(\overset{\to }{\rho }+m_{2}\mu^{-1}\overset{\to }{r}),$$

$$\overset{¨}{\overset{\to }{\rho }}=\frac{-M\mu^{-1}}{m_{3}}(\frac{F\left(r_{13}\right)}{r_{13}}(\overset{\to }{\rho }+m_{2}\mu^{-1}\overset{\to }{r})+\frac{F\left(r_{23}\right)}{r_{23}}\left(\overset{\to }{\rho }-m_{1}\mu^{-1}\overset{\to }{r}\right)).$$

### 2.1 Three identical $C_{70}$ fullerenes

The behaviour of $C_{70}$ fullerene seems like electron-deficient Alkenes and reacts rich with electron's species. The high symmetry exhibited by $C_{70}$ molecule has made it the unique property of the group. Carbon 70 fullerene's rotation around an axis in a plane has 120 symmetry operations which enable the mapping of its molecule onto itself, which in turn makes $C_{70}$ like $C_{60}$ the molecule with the most significant number of symmetry operations and the most symmetric molecules [6].

Given that the three $C_{70}$ fullerenes are moving uniformly in circular orbits with the same angular velocities in the same plane; we denote $\left(x_{i},y_{i},0\right)$ as the coordinates of the *i*−fullerenes. Then making use of equation (6), we obtain

(9) $$\overset{¨}{x_{k}}=m_{k}^{-1}\sum_{j\neq k}\frac{F\left(r_{jk}\right)}{r_{jk}}(x_{j}-x_{k}),$$

$$\overset{¨}{y_{k}}=m_{k}^{-1}\sum_{j\neq k}\frac{F\left(r_{jk}\right)}{r_{jk}}(y_{j}-y_{k}).$$

Given that, the angular velocity *ω*, the rotating coordinate system is as thus;

(10) $$x_{k}=\xi_{k}\cos\omega t-\eta_{k}\sin\omega t,$$

$$y_{k}=\xi_{k}\sin\omega t+\eta_{k}\cos\omega t.$$

From which we obtain,

(11) $$\overset{¨}{x_{k}}=\overset{¨}{\xi_{k}}-2\omega \overset{˙}{\eta_{k}}-\omega^{2}\xi_{k},$$

$$\overset{¨}{y_{k}}=\overset{¨}{\eta_{k}}+2\omega \overset{˙}{\xi_{k}}-\omega^{2}\eta_{k}.$$

Considering equations (10) and (11) in (9), we have

(12) $$\overset{¨}{\xi_{k}}-2\omega \overset{˙}{\eta_{k}}-\omega^{2}\xi_{k}=m_{k}^{-1}\sum_{j\neq k}\frac{F\left(r_{jk}\right)}{r_{jk}}(\xi_{j}-\xi_{k}),$$

$$\overset{¨}{\eta_{k}}+2\omega \overset{˙}{\xi_{k}}-\omega^{2}\eta_{k}=m_{k}^{-1}\sum_{j\neq k}\frac{F\left(r_{jk}\right)}{r_{jk}}(\eta_{j}-\eta_{k}).$$

The three fullerenes are assumed to be identical and assigned to the vertices of an equilateral triangle. i.e.; $m_{1}=m_{2}=m_{3}=m,\mu =m_{1}+m_{2}=2m,r_{12}=r_{13}=r_{23}=r$ and on assuming $F\left(r_{12}\right)=F\left(r_{13}\right)=F\left(r_{23}\right)=F\left(r\right)$, the first equation of (8) yields

(13) $$\overset{¨}{\overset{\to }{r}}=\frac{-3F(r)\overset{\to }{r}}{mr}.$$

Given that the angular velocity $\omega =\sqrt{\frac{3F(r)}{mr}}$, Eq. (13) represents the simple harmonic motion.

Because the attractive Van der Waals forces between molecules are the only source of centripetal forces between three fullerenes, by using equations (2), (5) and (13), we can predict the collective potential function (Φ(r)), mutual force (F(r)) and angular velocity (*ω*), shown in Table 2 and plotted in Figure 4, Figure 5, Figure 6, Figure 7.

## 3 Results

In this section, we have provided the results of the study both analytically and numerically. The analytical works are calculated based on the positions and stability natures of the research in question. The numerical works are computed with the help of the software MATHEMATICA and are presented in tables and graphs.

### 3.1 Positions of collinear equilibrium points of $C_{70}$ fullerene

We adopt a Cartesian coordinate system (0,ξ,η) spinning with angular velocity *ω* and the origin at the centre of mass of the two fullerenes to determine the position of the $C_{70}$ fullerene (test particle) with reference to the rotating frame under the effect of the other two $C_{70}$ fullerenes (primaries).

The positions of the two fullerenes are $\left(-\frac{R}{2},0\right)$ and $\left(\frac{R}{2},0\right)$ where *R* is the distance between them. The equations of motion of the $C_{70}$ fullerene under those as mentioned earlier circular planar assumptions can be obtained from equation (12) as

(14) $$\overset{¨}{\xi }-2\omega \overset{˙}{\eta }-\omega^{2}\xi =\frac{1}{m_{c}}(\frac{F\left(\rho_{1}\right)}{\rho_{1}}(-\frac{R}{2}-\xi )+\frac{F\left(\rho_{2}\right)}{\rho_{2}}(\frac{R}{2}-\xi )),$$

$$\overset{¨}{\eta }+2\omega \overset{˙}{\xi }-\omega^{2}\eta =\frac{1}{m_{c}}(\frac{F\left(\rho_{1}\right)}{\rho_{1}}(0-\eta )+\frac{F\left(\rho_{2}\right)}{\rho_{2}}(0-\eta )).$$

Where (ξ,η) are coordinates of the $C_{70}$ fullerene of mass $m_{c}$ and $\rho_{1}=\sqrt{{\left(\xi +\frac{R}{2}\right)}^{2}+\eta^{2}}$, $\rho_{2}=\sqrt{{\left(\xi -\frac{R}{2}\right)}^{2}+\eta^{2}}$ are its distances from the two fullerenes.

That is

(15) $$\overset{¨}{\xi }-2\omega \overset{˙}{\eta }=\frac{1}{2m_{c}}\frac{\partial \Phi }{\partial \xi },$$

$$\overset{¨}{\eta }+2\omega \overset{˙}{\xi }=\frac{1}{2m_{c}}\frac{\partial \Phi }{\partial \eta }.$$

Where $\Phi =\Phi_{1}+\Phi_{2}$ and $\Phi_{1}=\Psi \left(\rho \right)+m_{c}\omega^{2}\xi^{2}$, $\Phi_{2}=\Psi \left(\rho \right)+m_{c}\omega^{2}\eta^{2}$.

We now set $\overset{˙}{\xi }=\overset{˙}{\eta }=0=\overset{¨}{\xi }=\overset{¨}{\eta }$ in equation (15) and thus solve equations $\frac{\partial \Phi }{\partial \xi }=0=\frac{\partial \Phi }{\partial \eta }$ for *ξ* and *η*. Given that;

(16) $$\Phi =\frac{-A}{{\left({\left(\xi +\frac{R}{2}\right)}^{2}+\eta^{2}\right)}^{3}}+\frac{B}{{\left({\left(\xi +\frac{R}{2}\right)}^{2}+\eta^{2}\right)}^{6}}-\frac{A}{{\left({\left(\xi -\frac{R}{2}\right)}^{2}+\eta^{2}\right)}^{3}}+\frac{B}{{\left({\left(\xi -\frac{R}{2}\right)}^{2}+\eta^{2}\right)}^{6}}+m_{c}\omega^{2}(\xi^{2}+\eta^{2}).$$

Taking partial derivatives for *ξ* and *η* in equation (16) yields, respectively, equation (17) and (18).

(17) $$\frac{\partial \Phi }{\partial \xi }=\frac{-12B(\xi -\frac{R}{2})}{{\left(\eta^{2}+{\left(\xi -\frac{R}{2}\right)}^{2}\right)}^{7}}+\frac{6A(\xi -\frac{R}{2})}{{\left(\eta^{2}+{\left(\xi -\frac{R}{2}\right)}^{2}\right)}^{4}}-\frac{12B(\xi +\frac{R}{2})}{{\left(\eta^{2}+{\left(\xi +\frac{R}{2}\right)}^{2}\right)}^{7}}+\frac{6A(\xi +\frac{R}{2})}{{\left(\eta^{2}+{\left(\xi +\frac{R}{2}\right)}^{2}\right)}^{4}}+2\xi m_{c}\omega^{2}.$$

(18) $$\frac{\partial \Phi }{\partial \eta }=\frac{-12B\eta }{{\left(\eta^{2}+{\left(\xi -\frac{R}{2}\right)}^{2}\right)}^{7}}+\frac{6A\eta }{{\left(\eta^{2}+{\left(\xi -\frac{R}{2}\right)}^{2}\right)}^{4}}-\frac{12B\eta }{{\left(\eta^{2}+{\left(\xi +\frac{R}{2}\right)}^{2}\right)}^{7}}+\frac{6A\eta }{{\left(\eta^{2}+{\left(\xi +\frac{R}{2}\right)}^{2}\right)}^{4}}+2\eta m_{c}\omega^{2}.$$

At stationary points $\frac{\partial \Phi }{\partial \xi }=\frac{\partial \Phi }{\partial \eta }=0$ that is

(19) $$\frac{-12B(\xi -\frac{R}{2})}{{\left(\eta^{2}+{\left(\xi -\frac{R}{2}\right)}^{2}\right)}^{7}}+\frac{6A(\xi -\frac{R}{2})}{{\left(\eta^{2}+{\left(\xi -\frac{R}{2}\right)}^{2}\right)}^{4}}-\frac{12B\left(\xi +\frac{R}{2}\right)}{{\left(\eta^{2}+{\left(\xi +\frac{R}{2}\right)}^{2}\right)}^{7}}+\frac{6A\left(\xi +\frac{R}{2}\right)}{{\left(\eta^{2}+{\left(\xi +\frac{R}{2}\right)}^{2}\right)}^{4}}+2\xi m_{c}\omega^{2}=0.$$

(20) $$\frac{-12B\eta }{{\left(\eta^{2}+{\left(\xi -\frac{R}{2}\right)}^{2}\right)}^{7}}+\frac{6A\eta }{{\left(\eta^{2}+{\left(\xi -\frac{R}{2}\right)}^{2}\right)}^{4}}-\frac{12B\eta }{{\left(\eta^{2}+{\left(\xi +\frac{R}{2}\right)}^{2}\right)}^{7}}+\frac{6A\eta }{{\left(\eta^{2}+{\left(\xi +\frac{R}{2}\right)}^{2}\right)}^{4}}+2\eta m_{c}\omega^{2}=0.$$

With $A=17.411,B=29.011\times 10^{3},R=10.511,m_{c}=2.004\times 10^{-26},\omega =6.011\times 10^{11}$, equations (19) and (20) will reduce to

(21) $$0.0144818\xi -\frac{348132(-5.2555+\xi )}{{\left(\eta^{2}+{\left(-5.2555+\xi \right)}^{2}\right)}^{7}}+\frac{104.466(-5.2555+\xi )}{{\left(\eta^{2}+{\left(-5.2555+\xi \right)}^{2}\right)}^{4}}-\frac{348132\left(5.2555+\xi \right)}{{\left(\eta^{2}+{\left(5.2555+\xi \right)}^{2}\right)}^{7}}+\frac{104.466\left(5.2555+\xi \right)}{{\left(\eta^{2}+{\left(5.2555+\xi \right)}^{2}\right)}^{4}}=0.$$

(22) $$0.0144818\eta -\frac{348132\eta }{{\left(\eta^{2}+{\left(-5.2555+\xi \right)}^{2}\right)}^{7}}+\frac{104.466\eta }{{\left(\eta^{2}+{\left(-5.2555+\xi \right)}^{2}\right)}^{4}}-\frac{348132\eta }{{\left(\eta^{2}+{\left(5.2555+\xi \right)}^{2}\right)}^{7}}+\frac{104.466\eta }{{\left(\eta^{2}+{\left(5.2555+\xi \right)}^{2}\right)}^{4}}=0.$$

The positions of stationary points of fullerenes are illustrated in Table 3 and Figure 8, Figure 9, Figure 10, Figure 11 using the software MATHEMATICA.

### 3.2 Stability of collinear equilibrium points of $C_{70}$ fullerene

Other types of problems can be solved using the concept of stability. It is most likely a crucial concept in science because it refers to what we refer to as “reality.” To be observable, everything should be stable. Energy levels, for example, are stable in quantum mechanics because unstable levels are not visible. If the autonomous system of differential equations $\overset{˙}{x}=X(x)$ is written in the form $\overset{˙}{x}=Ax+f(x)$ then the linearized system of equations is $\overset{˙}{x}=Ax$. Here, A is a constant matrix and f(x) is a vector function such that $\frac{f(x)}{\left|x\right|}\to 0$ as |x|→0 for t≥0. This requirement on f(x) is satisfied, for instance, when the components of f(x) are convergent power series beginning with second-order terms in *x*. The stability properties for $t\geq t_{0}$ of the linearized system may be stated simply:

(a) For complex roots of the characteristic equation of A, we have the following properties:
  i. When the characteristic roots all have negative real parts, the equilibrium point is asymptotically stable. This is true also when some of the roots are multiple.
  ii. When some or all of the characteristic roots have positive real parts, the equilibrium point is unstable. This is also true when some of the roots are multiple.
(b) For pure imaginary roots, the motion is oscillatory, and the solution is stable though it is not asymptotically stable. If there are multiple roots, the solution contains mixed (periodic and secular) terms, and the equilibrium point is unstable.

If all the roots are real and all negative, the solution is stable; if any of the roots are positive, the point is unstable. These statements are also valid for multiple roots.

By establishing the characteristic equation of the system under study, we can investigate the stability of a $C_{70}$ fullerene's motion in the vicinity of two $C_{70}$ fullerenes.

To obtain the variational equations, we denote the position of a stationary point of a $C_{70}$ fullerene by $\left(\xi_{o},\eta_{o}\right)$ and then assume the small displacements in $\left(\xi_{o},\eta_{o}\right)$ to be (α,θ) and writing Ω for Φ in equation (15). That is;

$$\xi =\xi_{o}+\alpha \text{ and }\eta =\eta_{o}+\theta ;$$

Taking the derivative, we have

(23) $$\overset{˙}{\xi }=\overset{˙}{\alpha },\;\overset{¨}{\xi }=\overset{¨}{\alpha };$$

$$\overset{˙}{\eta }=\overset{˙}{\theta },\;\overset{¨}{\eta }=\overset{¨}{\theta }.$$

Substituting equations (23) into equation (15) gives the variational equations of motion:

(24) $$\overset{¨}{\alpha }-2\omega \overset{˙}{\theta }=\frac{1}{2m_{c}}\left[\left(\Omega_{\xi \xi }^{0}\right)\alpha +(\Omega_{\xi \eta }^{0})\theta \right],$$

$$\overset{¨}{\theta }+2\omega \overset{˙}{\alpha }=\frac{1}{2m_{c}}\left[\left(\Omega_{\eta \xi }^{0}\right)\alpha +(\Omega_{\eta \eta }^{0})\theta \right].$$

The partial derivatives are evaluated at the point under consideration, as indicated by the superscript o.

Assume;

$$\alpha =k_{1}e^{\lambda t},\;\theta =k_{2}e^{\lambda t}\text{ then},$$

(25) $$\overset{˙}{\alpha }=k_{1}\lambda e^{\lambda t},\;\overset{˙}{\theta }=k_{2}\lambda e^{\lambda t},$$

$$\overset{¨}{\alpha }=k_{1}\lambda^{2}e^{\lambda t},\;\overset{¨}{\theta }=k_{2}\lambda^{2}e^{\lambda t}.$$

Making use of equations (25) into (24), we have

(26) $$\left(2m_{c}\lambda^{2}-\Omega_{\xi \xi }^{0}\right)k_{1}+\left(-4\omega m_{c}\lambda -\Omega_{\xi \eta }^{0}\right)k_{2}=0,$$

$$\left(4\omega m_{c}\lambda -\Omega_{\eta \xi }^{0}\right)k_{1}+\left(2m_{c}\lambda^{2}-\Omega_{\eta \eta }^{0}\right)k_{2}=0,$$

which results in a nontrivial solution if

$$\left|\begin{array}{cc} 2m_{c}\lambda^{2}-\Omega_{\xi \xi }^{0}\; & -4\omega m_{c}\lambda -\Omega_{\xi \eta }^{0}\\ 4\omega m_{c}\lambda -\Omega_{\eta \xi }^{0}\; & 2m_{c}\lambda^{2}-\Omega_{\eta \eta }^{0} \end{array}\right|=0,$$

expanding the determinant, we obtain

(27) $$\lambda^{4}-\left[\frac{1}{2m_{c}}\left(\Omega_{\xi \xi }^{0}+\Omega_{\eta \eta }^{0}-8\omega^{2}m_{c}\right)\right]\lambda^{2}+\left[\frac{\omega }{m_{c}}\left(\Omega_{\xi \eta }^{0}-\Omega_{\eta \xi }^{0}\right)\right]\lambda +\left[\frac{1}{4m_{c}^{2}}\left(\Omega_{\xi \xi }^{0}\Omega_{\eta \eta }^{0}-\Omega_{\xi \eta }^{0}\Omega_{\eta \xi }^{0}\right)\right]=0.$$

Equation (27) is the characteristic equation for the stated problem.

From equations (17) and (18), the first-order partial derivatives of Ω (on changing Φ into Ω) are;

(28) $$\Omega_{\xi }=\frac{-12B(\xi -\frac{R}{2})}{{\left(\eta^{2}+{\left(\xi -\frac{R}{2}\right)}^{2}\right)}^{7}}+\frac{6A(\xi -\frac{R}{2})}{{\left(\eta^{2}+{\left(\xi -\frac{R}{2}\right)}^{2}\right)}^{4}}-\frac{12B(\xi +\frac{R}{2})}{{\left(\eta^{2}+{\left(\xi +\frac{R}{2}\right)}^{2}\right)}^{7}}+\frac{6A(\xi +\frac{R}{2})}{{\left(\eta^{2}+{\left(\xi +\frac{R}{2}\right)}^{2}\right)}^{4}}+2\xi m_{c}\omega^{2}.$$

(29) $$\Omega_{\eta }=\frac{-12B\eta }{{\left(\eta^{2}+{\left(\xi -\frac{R}{2}\right)}^{2}\right)}^{7}}+\frac{6A\eta }{{\left(\eta^{2}+{\left(\xi -\frac{R}{2}\right)}^{2}\right)}^{4}}-\frac{12B\eta }{{\left(\eta^{2}+{\left(\xi +\frac{R}{2}\right)}^{2}\right)}^{7}}+\frac{6A\eta }{{\left(\eta^{2}+{\left(\xi +\frac{R}{2}\right)}^{2}\right)}^{4}}+2\eta m_{c}\omega^{2}.$$

The values of the second-order partial derivatives at the stationary point under study are calculated using equations (28) and (29).

(30) $$\Omega_{\xi \xi }^{0}=\frac{6A}{{\left(\eta_{o}^{2}+{\left(\xi_{o}-\frac{R}{2}\right)}^{2}\right)}^{4}}-\frac{12B}{{\left(\eta_{o}^{2}+{\left(\xi_{o}-\frac{R}{2}\right)}^{2}\right)}^{7}}-\frac{12B}{{\left(\eta_{o}^{2}+{\left(\xi_{o}+\frac{R}{2}\right)}^{2}\right)}^{7}}+\frac{6A}{{\left(\eta_{o}^{2}+{\left(\xi_{o}+\frac{R}{2}\right)}^{2}\right)}^{4}}+\frac{168B{\left(\xi_{o}-\frac{R}{2}\right)}^{2}}{{\left(\eta_{o}^{2}+{\left(\xi_{o}-\frac{R}{2}\right)}^{2}\right)}^{8}}-\frac{48A{\left(\xi_{o}-\frac{R}{2}\right)}^{2}}{{\left(\eta_{o}^{2}+{\left(\xi_{o}-\frac{R}{2}\right)}^{2}\right)}^{5}}+\frac{168B{\left(\xi_{o}+\frac{R}{2}\right)}^{2}}{{\left(\eta_{o}^{2}+{\left(\xi_{o}+\frac{R}{2}\right)}^{2}\right)}^{8}}-\frac{48A{\left(\xi_{o}+\frac{R}{2}\right)}^{2}}{{\left(\eta_{o}^{2}+{\left(\xi_{o}+\frac{R}{2}\right)}^{2}\right)}^{5}}+2m_{c}\omega^{2}.$$

(31) $$\Omega_{\eta \eta }^{0}=\frac{168B\eta_{o}^{2}}{{\left(\eta_{o}^{2}+{\left(\xi_{o}-\frac{R}{2}\right)}^{2}\right)}^{8}}-\frac{12B}{{\left(\eta_{o}^{2}+{\left(\xi_{o}-\frac{R}{2}\right)}^{2}\right)}^{7}}-\frac{48A\eta_{o}^{2}}{{\left(\eta_{o}^{2}+{\left(\xi_{o}-\frac{R}{2}\right)}^{2}\right)}^{5}}+\frac{6A}{{\left(\eta_{o}^{2}+{\left(\xi_{o}-\frac{R}{2}\right)}^{2}\right)}^{4}}+\frac{168B\eta_{o}^{2}}{{\left(\eta_{o}^{2}+{\left(\xi_{o}+\frac{R}{2}\right)}^{2}\right)}^{8}}-\frac{12B}{{\left(\eta_{o}^{2}+{\left(\xi_{o}+\frac{R}{2}\right)}^{2}\right)}^{7}}-\frac{48A\eta_{o}^{2}}{{\left(\eta_{o}^{2}+{\left(\xi_{o}+\frac{R}{2}\right)}^{2}\right)}^{5}}+\frac{6A}{{\left(\eta_{o}^{2}+{\left(\xi_{o}+\frac{R}{2}\right)}^{2}\right)}^{4}}+2m_{c}\omega^{2},$$

and

(32) $$\Omega_{\xi \eta }^{0}=\frac{168B\eta_{o}(\xi_{o}-\frac{R}{2})}{{\left(\eta_{o}^{2}+{\left(\xi_{o}-\frac{R}{2}\right)}^{2}\right)}^{8}}-\frac{48A\eta_{o}\left(\xi_{o}-\frac{R}{2}\right)}{{\left(\eta_{o}^{2}+{\left(\xi_{o}-\frac{R}{2}\right)}^{2}\right)}^{5}}+\frac{168B\eta_{o}(\xi_{o}+\frac{R}{2})}{{\left(\eta_{o}^{2}+{\left(\xi_{o}+\frac{R}{2}\right)}^{2}\right)}^{8}}-\frac{48A\eta_{o}(\xi_{o}+\frac{R}{2})}{{\left(\eta_{o}^{2}+{\left(\xi_{o}+\frac{R}{2}\right)}^{2}\right)}^{5}}.$$

(33) $$\Omega_{\eta \xi }^{0}=\frac{168B\eta_{o}(\xi_{o}-\frac{R}{2})}{{\left(\eta_{o}^{2}+{\left(\xi_{o}-\frac{R}{2}\right)}^{2}\right)}^{8}}-\frac{48A\eta_{o}\left(\xi_{o}-\frac{R}{2}\right)}{{\left(\eta_{o}^{2}+{\left(\xi_{o}-\frac{R}{2}\right)}^{2}\right)}^{5}}+\frac{168B\eta_{o}(\xi_{o}+\frac{R}{2})}{{\left(\eta_{o}^{2}+{\left(\xi_{o}+\frac{R}{2}\right)}^{2}\right)}^{8}}-\frac{48A\eta_{o}(\xi_{o}+\frac{R}{2})}{{\left(\eta_{o}^{2}+{\left(\xi_{o}+\frac{R}{2}\right)}^{2}\right)}^{5}}.$$

Equations (32) and (33) are equal. That is $\Omega_{\xi \eta }^{0}=\Omega_{\eta \xi }^{0}$. Therefore, equation (27) becomes

(34) $$\lambda^{4}-\left[\frac{1}{2m_{c}}\left(\Omega_{\xi \xi }^{0}+\Omega_{\eta \eta }^{0}-8\omega^{2}m_{c}\right)\right]\lambda^{2}+\left[\frac{1}{4m_{c}^{2}}\left(\Omega_{\xi \xi }^{0}\Omega_{\eta \eta }^{0}-{\left(\Omega_{\xi \eta }^{0}\right)}^{2}\right)\right]=0.$$

Rewriting (34), we have

(35) $$\lambda^{4}-\left[\frac{1}{2m_{c}}\left(K+L-8\omega^{2}m_{c}\right)\right]\lambda^{2}+\left[\frac{1}{4m_{c}^{2}}\left(KL-M^{2}\right)\right]=0,$$

where;

$$K=\frac{6A}{{\left(\eta_{o}^{2}+{\left(\xi_{o}-\frac{R}{2}\right)}^{2}\right)}^{4}}-\frac{12B}{{\left(\eta_{o}^{2}+{\left(\xi_{o}-\frac{R}{2}\right)}^{2}\right)}^{7}}-\frac{12B}{{\left(\eta_{o}^{2}+{\left(\xi_{o}+\frac{R}{2}\right)}^{2}\right)}^{7}}+\frac{6A}{{\left(\eta_{o}^{2}+{\left(\xi_{o}+\frac{R}{2}\right)}^{2}\right)}^{4}}+\frac{168B{\left(\xi_{o}-\frac{R}{2}\right)}^{2}}{{\left(\eta_{o}^{2}+{\left(\xi_{o}-\frac{R}{2}\right)}^{2}\right)}^{8}}-\frac{48A{\left(\xi_{o}-\frac{R}{2}\right)}^{2}}{{\left(\eta_{o}^{2}+{\left(\xi_{o}-\frac{R}{2}\right)}^{2}\right)}^{5}}+\frac{168B{\left(\xi_{o}+\frac{R}{2}\right)}^{2}}{{\left(\eta_{o}^{2}+{\left(\xi_{o}+\frac{R}{2}\right)}^{2}\right)}^{8}}-\frac{48A{\left(\xi_{o}+\frac{R}{2}\right)}^{2}}{{\left(\eta_{o}^{2}+{\left(\xi_{o}+\frac{R}{2}\right)}^{2}\right)}^{5}}+2m_{c}\omega^{2},$$

$$L=\frac{168B\eta_{o}^{2}}{{\left(\eta_{o}^{2}+{\left(\xi_{o}-\frac{R}{2}\right)}^{2}\right)}^{8}}-\frac{12B}{{\left(\eta_{o}^{2}+{\left(\xi_{o}-\frac{R}{2}\right)}^{2}\right)}^{7}}-\frac{48A\eta_{o}^{2}}{{\left(\eta_{o}^{2}+{\left(\xi_{o}-\frac{R}{2}\right)}^{2}\right)}^{5}}+\frac{6A}{{\left(\eta_{o}^{2}+{\left(\xi_{o}-\frac{R}{2}\right)}^{2}\right)}^{4}}+\frac{168B\eta_{o}^{2}}{{\left(\eta_{o}^{2}+{\left(\xi_{o}+\frac{R}{2}\right)}^{2}\right)}^{8}}-\frac{12B}{{\left(\eta_{o}^{2}+{\left(\xi_{o}+\frac{R}{2}\right)}^{2}\right)}^{7}}-\frac{48A\eta_{o}^{2}}{{\left(\eta_{o}^{2}+{\left(\xi_{o}+\frac{R}{2}\right)}^{2}\right)}^{5}}+\frac{6A}{{\left(\eta_{o}^{2}+{\left(\xi_{o}+\frac{R}{2}\right)}^{2}\right)}^{4}}+2m_{c}\omega^{2}\text{ and}$$

$$M=\frac{168B\eta_{o}(\xi_{o}-\frac{R}{2})}{{\left(\eta_{o}^{2}+{\left(\xi_{o}-\frac{R}{2}\right)}^{2}\right)}^{8}}-\frac{48A\eta_{o}\left(\xi_{o}-\frac{R}{2}\right)}{{\left(\eta_{o}^{2}+{\left(\xi_{o}-\frac{R}{2}\right)}^{2}\right)}^{5}}+\frac{168B\eta_{o}(\xi_{o}+\frac{R}{2})}{{\left(\eta_{o}^{2}+{\left(\xi_{o}+\frac{R}{2}\right)}^{2}\right)}^{8}}-\frac{48A\eta_{o}(\xi_{o}+\frac{R}{2})}{{\left(\eta_{o}^{2}+{\left(\xi_{o}+\frac{R}{2}\right)}^{2}\right)}^{5}}.$$

### 3.3 Numerical applications

Equations (21), (22) and (35) are used to calculate the locations and stability of the stationary points, as well as the $C_{70}$ fullerene in the vicinity of two $C_{70}$ fullerenes, using a software package called MATHEMATICA. These computations are done with the numerical data of the parameters used in the model, as presented in Table 1. Table 3, Figure 8, Figure 9, Figure 10, and Table 4 depict the positions and stability of the above-mentioned problem. Table 4 shows that for each set of values with a positive real part, there is at least one complex root. As a result, they are unstable in a Lyapunov sense. This confirms the works of [16], [17], [18], [19], [20], [21], [22].

**Table 1:** Numerical data used in the model.

S/NO.	CONSTANT NAME	SYMBOL	VALUE
1.	Radius of *C*_70_	*a*	$3.561\;{}_{A}^{o}$
2.	Carbon-Carbon bond length	*φ*	$1.432\;{}_{A}^{o}$
3.	Mass of a single carbon atom	*m*_*c*_	2.004 × 10^−26^ kg
4.	Mean surface density of *C*_70_ fullerene	*n*_*f*_	$0.3889\;{}_{A^{-2}}^{o}$
5.	Mean surface density of Graphene	*n*_*g*_	$0.3812\;{}_{A^{-2}}^{o}$
6.	Mass of a single *C*_70_ fullerene	*m*	1.207 × 10^−24^ kg
7.	Attractive constant	*A*	$17.411\;\text{eV}\times \;{}_{A^{6}}^{o}$
8.	Repulsive constant	*B*	29.011 × 10^3^ eV × 10^12^

## 4 Discussion

This paper investigates the classical restricted three-body problem of three carbon 70 fullerenes ($C_{70}$ fullerenes) at the nanoscale. Analytically, we determined the positions and stability of the $C_{70}$ fullerene. Numerically, we have computed the positions and stability of $C_{70}$ fullerene at the nanoscale to show the effects of the parameters involved with the help of MATHEMATICA.

**Figure 1:**
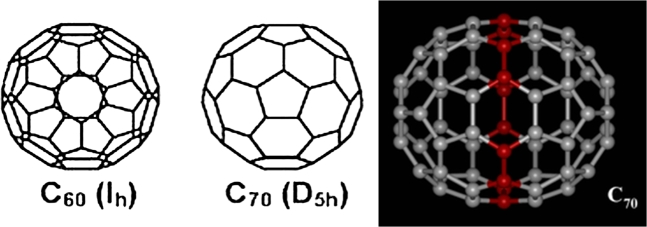
The structure of the C_60 and_ C_70_ molecule. Red atoms indicate the five additional hexagons to the C_60_ molecule.

**Figure 2:**
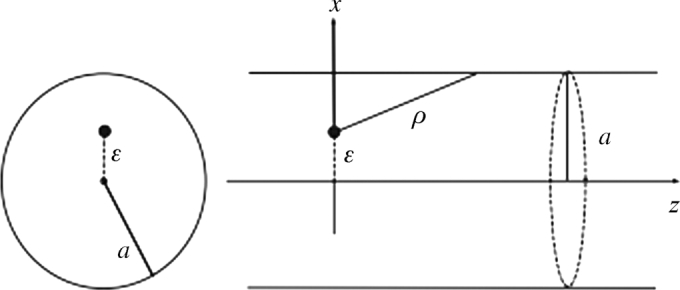
An offset atom in a carbon nanotube.

**Figure 3:**
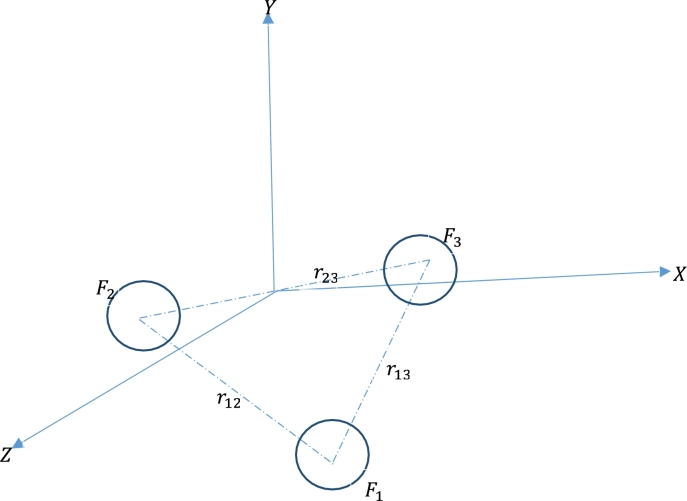
Three-body problem at the nanoscale.

The structure of $C_{70}$ fullerenes, as shown in Fig. 1, has similar properties with the $C_{60}$ molecule but has a belt of 5 hexagons inserted at the equator. We have investigated these properties considering the Lennard-Jones potential energy for an offset atom and an offset fullerene, as shown in Fig. 2. The motion of the three-body problem of the model under consideration is demonstrated in Fig. 3.

**Source:**http://fullerenes materials/Elias lectures chemistry of carbon fullerenes sept. 2015.pdf.

**Source:**http://rspa.royalsocietypublishing.org/content/463/2078/477.full.html#ref-list-1.

**Figure 4:**
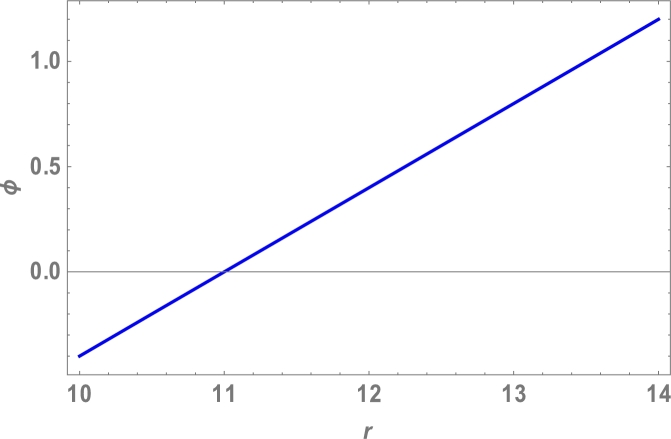
Effects of collective Potential Function and Distance between two typical surface elements.

**Figure 5:**
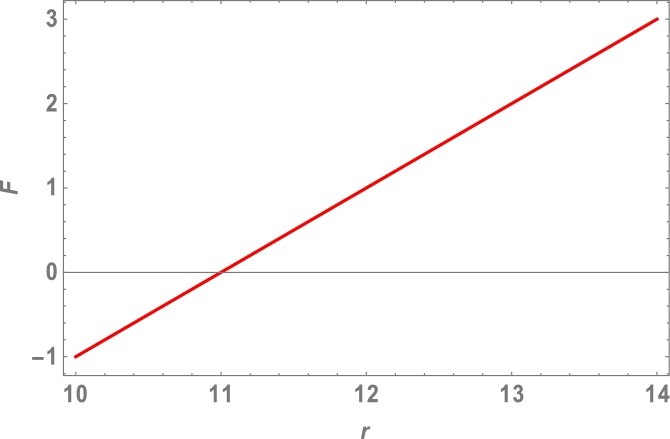
Effects of collective Mutual Force and Distance between two typical surface elements.

**Figure 6:**
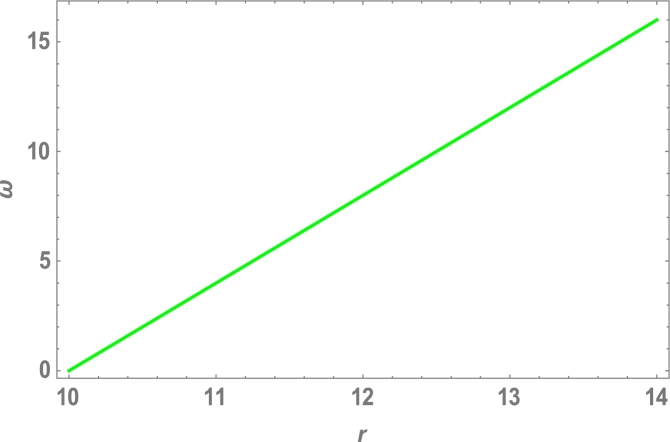
Effects of collective Angular Velocity and Distance between two typical surface elements.

**Figure 7:**
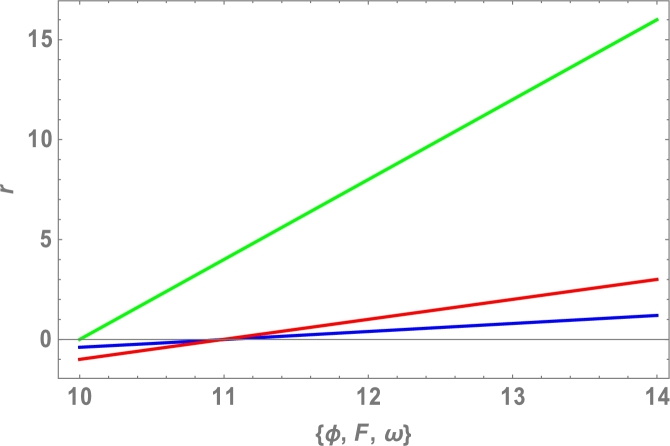
Effects of collective Potential Function, Mutual Force, Angular Velocity and Distance between two typical surface elements.

The total molecular energy is defined as the sum of all pairwise molecular interactions between two $C_{70}$ fullerenes, which is an approximation of a continuous approach as shown in equation (1). We expected the collective potential function, mutual force, and angular velocity, as shown in Table 2 and Figure 4, Figure 5, Figure 6, Figure 7, since the centripetal forces between three fullerenes are provided by attractive Vand der Waals forces between only molecules.

**Table 2:** Attractive Van der Waals forces between molecules.

$r({}_{A}^{o})$	Φ(r)(eV)	F(r)(N)	*ω* (rad)
10	−0.4	−1.0	0
10.5	−0.2	−0.5	2
11	0	0	4
11.5	0.2	0.5	6
12	0.4	1.0	8
12.5	0.6	1.5	10
13	0.8	2.0	12
13.5	1.0	2.5	14
14	1.2	3.0	16

**Figure 8:**
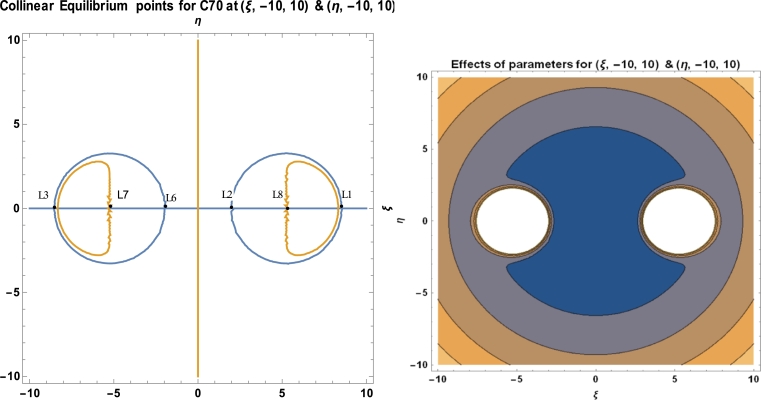
Positions of collinear equilibrium points of *C*_70_ fullerene for (ξ,−10,10)&(η,−10,10).

**Figure 9:**
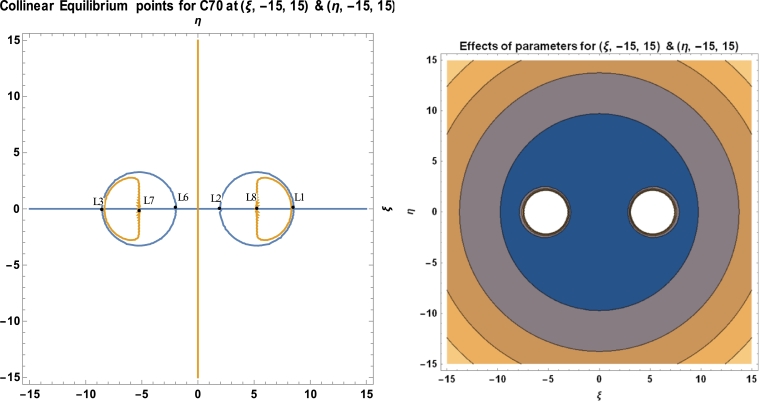
Positions of collinear equilibrium points of *C*_70_ fullerene for (ξ,−15,15)&(η,−15,15).

**Figure 10:**
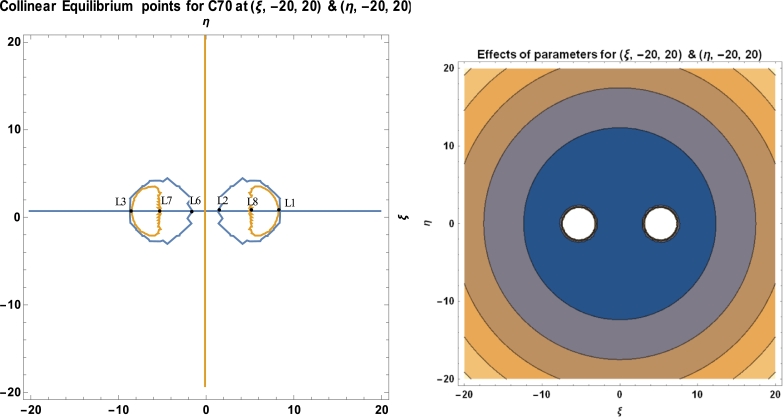
Positions of collinear equilibrium points of *C*_70_ fullerene for (ξ,−20,20)&(η,−20,20).

We have utilized the software MATHEMATICA in plotting our graphs. We observed that, for a given initial conditions {(ξ,−15,15),(η,−10,10)}, the positions of the stationary points of the fullerenes possessed two regular islands' region of attraction, both on the positive and negative sides of the axes (see Fig. 8). In Fig. 9, we witness irregular region of attraction of islands that seem to be compressed and very close to the origin with the initial conditions {(ξ,−30,30),(η,−20,20)}. The region of attraction becomes larger and more regular with initial conditions {(ξ,−7,8),(η,−7,8)} as represented in Fig. 10 above.

**Figure 11:**
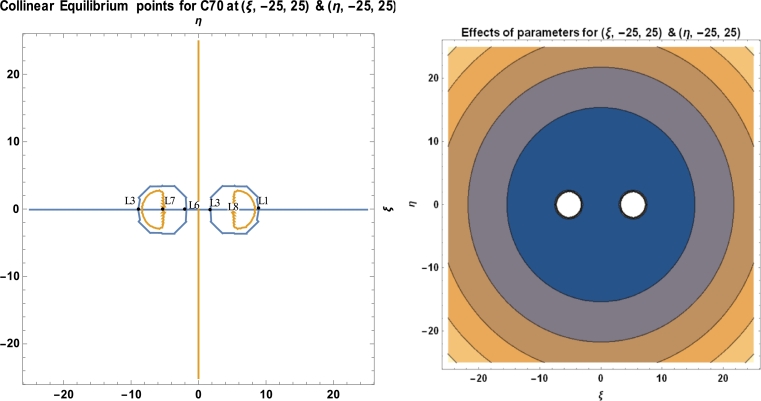
Positions of collinear equilibrium points of *C*_70_ fullerene for (ξ,−25,25)&(η,−25,25).

## 5 Conclusion

We have investigated the positions and stability of $C_{70}$ fullerenes at the nanoscale. The positions and stability of the problem under review are computed with the help of software MATHEMATICA to show the effects of the parameters involved.

As demonstrated in Figure 8, Figure 9, Figure 10, the positions of the stationary points of the $C_{70}$ fullerenes revealed that, for a bit of change in the initial conditions, the behaviour of the region of attraction changes accordingly. The region of attraction result in either irregular, which moves close to the origin or regular, away from the origin depending on the given initial conditions.

The positions of the stationary points, as evidenced in Table 3 and Figure 8, Figure 9, Figure 10, indicate that the points are collinearly, laying on the ξ−axis which are symmetric about the η−axis as the molecules of the carbon atom are evenly distributed about the nucleus. The collinear points, as shown in Figure 8, Figure 9, Figure 10, move closer and away from the origin as the range of the carbon atom increases and decreases, respectively.

**Table 3:** Positions of collinear equilibrium points of *C*_70_ fullerene.

*A*	*B*	*R*	*m*_*c*_	*ω*	*ξ*	±*η*
17.411	29.011 × 10^3^	10.511	2.004 × 10^−26^	6.011 × 10^11^	0	0
					−8.32645	0
					8.32645	0

As shown in Table 4, there is at least one complex root with a positive real part for each set of values. As a result, the stationary points are unstable in the Lyapunov sense.

**Table 4:** Stability of collinear equilibrium points of *C*_70_ fullerene for *A* = 17.411, *B* = 29.011 × 10^3^, *R* = 10.511, *m*_*c*_ = 2.004 × 10^−26^, *ω* = 6.011 × 10^11^.

*ξ*	±*η*	±*λ*_1,2_	±*λ*_3,4_
0	0	−2.27953 × 10^11^ ± 6.57005 × 10^11^*i*	2.27953 × 10^11^ ± 6.57005 × 10^11^*i*
−8.32645	0	±3.78323 × 10^12^*i*	0 ± 1.14125 × 10^12^*i*
8.32645	0	±3.78323 × 10^12^*i*	0 ± 1.14125 × 10^12^*i*

## Declarations

### Author contribution statement

Tyokyaa Kanshio Richard: Performed the experiments; Analyzed and interpreted the data; Wrote the paper.

Jagadish Singh: Conceived and designed the experiments; Contributed reagents, materials, analysis tools or data.

### Funding statement

This research did not receive any specific grant from funding agencies in the public, commercial, or not-for-profit sectors.

### Data availability statement

Data included in article/supplementary material/referenced in article.

### Declaration of interests statement

The authors declare no conflict of interest.

### Additional information

No additional information is available for this paper.
